# Oxytocin increases liking for a country's people and national flag but not for other cultural symbols or consumer products

**DOI:** 10.3389/fnbeh.2014.00266

**Published:** 2014-08-05

**Authors:** Xiaole Ma, Lizhu Luo, Yayuan Geng, Weihua Zhao, Qiong Zhang, Keith M. Kendrick

**Affiliations:** Social Cognitive and Affective Neuroscience, Key Laboratory for NeuroInformation of Ministry of Education, School of Life Science and Technology, University of Electronic Science and Technology of ChinaChengdu, China

**Keywords:** ethnocentrism, in-group, oxytocin, national flag, social

## Abstract

The neuropeptide oxytocin enhances in-group favoritism and ethnocentrism in males. However, whether such effects also occur in women and extend to national symbols and companies/consumer products is unclear. In a between-subject, double-blind placebo controlled experiment we have investigated the effect of intranasal oxytocin on likeability and arousal ratings given by 51 adult Chinese males and females for pictures depicting people or national symbols/consumer products from both strong and weak in-groups (China and Taiwan) and corresponding out-groups (Japan and South Korea). To assess duration of treatment effects subjects were also re-tested after 1 week. Results showed that although oxytocin selectively increased the bias for overall liking for Chinese social stimuli and the national flag, it had no effect on the similar bias toward other Chinese cultural symbols, companies, and consumer products. This enhanced bias was maintained 1 week after treatment. No overall oxytocin effects were found for Taiwanese, Japanese, or South Korean pictures. Our findings show for the first time that oxytocin increases liking for a nation's society and flag in both men and women, but not that for other cultural symbols or companies/consumer products.

## Introduction

Cultural bias and prejudice provides both the glue which binds large social groups together as well as laying the foundations for intergroup conflict. In recent years considerable interest has focused on the potential role of the hypothalamic neuropeptide oxytocin (OXT) in promoting such social biases in terms of strengthening in-group affiliations and derogation of out-groups. Previous studies have reported that intranasal OXT treatment facilitated parochial altruism in the context of economic games by enhancing trust and co-operation with in-group members and defensive aggression toward competing out-group ones (De Dreu et al., 2010; De Dreu, 2012). Oxytocin also promotes non-co-operation in intergroup conflict situations in the context of protecting vulnerable in-group members (De Dreu et al., 2012b). Intranasal OXT has been reported to increase ethnocentric behavior in economic tasks, human emotion attribution and decision making in moral dilemma tasks, although primarily by facilitating in-group favoritism rather than out-group derogation (De Dreu et al., 2011; De Dreu, 2012; Van IJzendoorn and Bakermans-Kranenburg, 2012). On the other hand empathy for pain experienced by individuals in an out-group has recently been found to be increased by OXT (Shamay-Tsoory et al., 2013), and one study has shown that it can increase co-operation with both in-groups and out-groups in non-competitive situations (Israel et al., 2012). Other studies have shown that OXT decreases generosity in similar economic tasks toward unknown players (Radke and de Bruijn, 2012) and biases choice toward “high-risk” allies (De Dreu et al., 2012a). However, all but one of these studies (De Dreu et al., 2012a) involved only male subjects and there is increasing evidence for both neural and gender differences in the effects of OXT (Kubzansky et al., 2012; Ditzen et al., 2013; Fischer-Shofty et al., 2013; Rilling et al., 2014; Yao et al., 2014). Previous studies have also not directly addressed whether OXT promotes ethnocentrism by increasing actual liking or disliking of either some or all categories of social cultural stimuli.

Ethnocentrism extends beyond people in different cultures to include attitudes toward a number of different categories of cultural iconic non-social symbols, such as flags, cities, buildings and monuments, money and food, and consumer products marketed internationally by national companies. In terms of consumer products this is known as the “country of origin effect,” and while a number of factors influence its strength, there is support for ethnocentric attitudes affecting purchasing behavior (Shimp and Sharma, 1987; Jiménez and Martín, 2010). No studies to date have investigated whether OXT influences this cultural symbol and consumer-product-based ethnocentrism.

In the current study we have therefore investigated the effect of intranasal OXT on the likes and dislikes of adult male and female Chinese subjects for pictures across a range of different social and non-social categories from their own (Chinese) and a closely related culture (Taiwanese) in comparison with those from other familiar Asian cultures with a long-standing history of either a mild (South Korean) or strong (Japanese) out-group derogation. We included countries Chinese people have either weak or strong positive or negative biases toward due to evidence that OXT-effects are often context-dependent (Bartz et al., 2011). To investigate whether OXT-induced behavioral effects were enduring, we re-tested subjects1 week after the initial experiment. In view of the fact that neither the categories of different social and non-social stimuli used can be considered as homogeneous, and that some non-social cultural categories have strong social associations (such as flags) we also investigated potential differential effects of OXT on different individual categories of social and non-social stimuli. We hypothesized that since other studies have shown OXT facilitates responses toward social but not non-social stimuli (Hurlemann et al., 2010; Meyer-Lindenberg et al., 2011; Striepens et al., 2011), it would increase “liking” of social stimuli from in-group cultures, but not for their non-social national symbols and consumer products. However, we also hypothesized that there might be some potential differences in OXT effects across the individual social and non-social categories as a result of contextual aspects of cultural experience. As a control for other potential non-specific effects of OXT subjects were also required to rate their arousal responses and familiarity for the stimulus pictures although we did not anticipate any effects on these parameters in line with many previous experiments (Striepens et al., 2011, 2014; Scheele et al., 2013). As a further control we also investigated potential contributions of the female menstrual cycle although once again in line with previous findings (Theodoridou et al., 2009; Domes et al., 2010) we did not anticipate any influence of this on OXT effects.

## Methods

A total of 51 male and female (female *n* = 26) Chinese (Han) subjects were included in the study. The subjects were all University students with an average age of mean ± s.e.m. = 22.16 ± 0.22 years and were free of medical or psychiatric illness, drug or alcohol abuse. None of the female subjects were taking the contraceptive pill and no attempt was made to control for stage of menstrual cycle other than in the first test where OXT and placebo (PLC) treatments and were not given to subjects during their menstrual period. On the basis of self-reports of menstruation dates, female subjects were divided into those estimated to be in the follicular phase or in the luteal phase of their cycle at the time of test 1, although the majority (19/26) were in the luteal phase. The study used a double-blind, between-subject, placebo-controlled design with subjects randomly assigned to OXT (male, *n* = 14; female, *n* = 13) and PLC (male, *n* = 11; female, *n* = 13) treatment groups and tested individually. The study was approved by the ethical committee of the University of Electronic Science and Technology of China and all subjects gave informed consent to take part.

Immediately before the experiment all subjects completed a range of questionnaires measuring personality and affective traits and levels of anxiety and depression: Chinese versions of: Beck Depression Inventory (BDI-II), Empathy Quotient (EQ), NEO-Five Factor Inventory (NEO-FFI), Positive and Negative Affect Schedule (PANAS), State-Trait Anxiety Inventory (STAI), and Self-Esteem Scale (SES).

For the experiment subjects were first administered a single intranasal dose of 24IU OXT (Syntocinon Spray—Sichuan Meike Pharmacy Co. Ltd, Sichuan, China; 3 puffs of 4 IU per nostril with 30 s between each puff) or PLC (also 3 puffs per nostril). The PLC treatment was also provided in the same type of dispenser bottle by the pharmaceutical supply company providing the OXT nasal spray, and contained all of same ingredients other than the neuropeptide. In line with many previous reports (Striepens et al., 2011; Guastella et al., 2013) the experimental paradigm started 45 min after OXT or PLC treatment which is estimated to allow maximum increased concentrations of the peptide to occur within the cerebrospinal fluid (Born et al., 2002; Chang et al., 2012; Striepens et al., 2013). In post-experiment interviews subjects were unable to identify better than chance whether they had received the OXT or PLC treatment.

**Figure 1:**
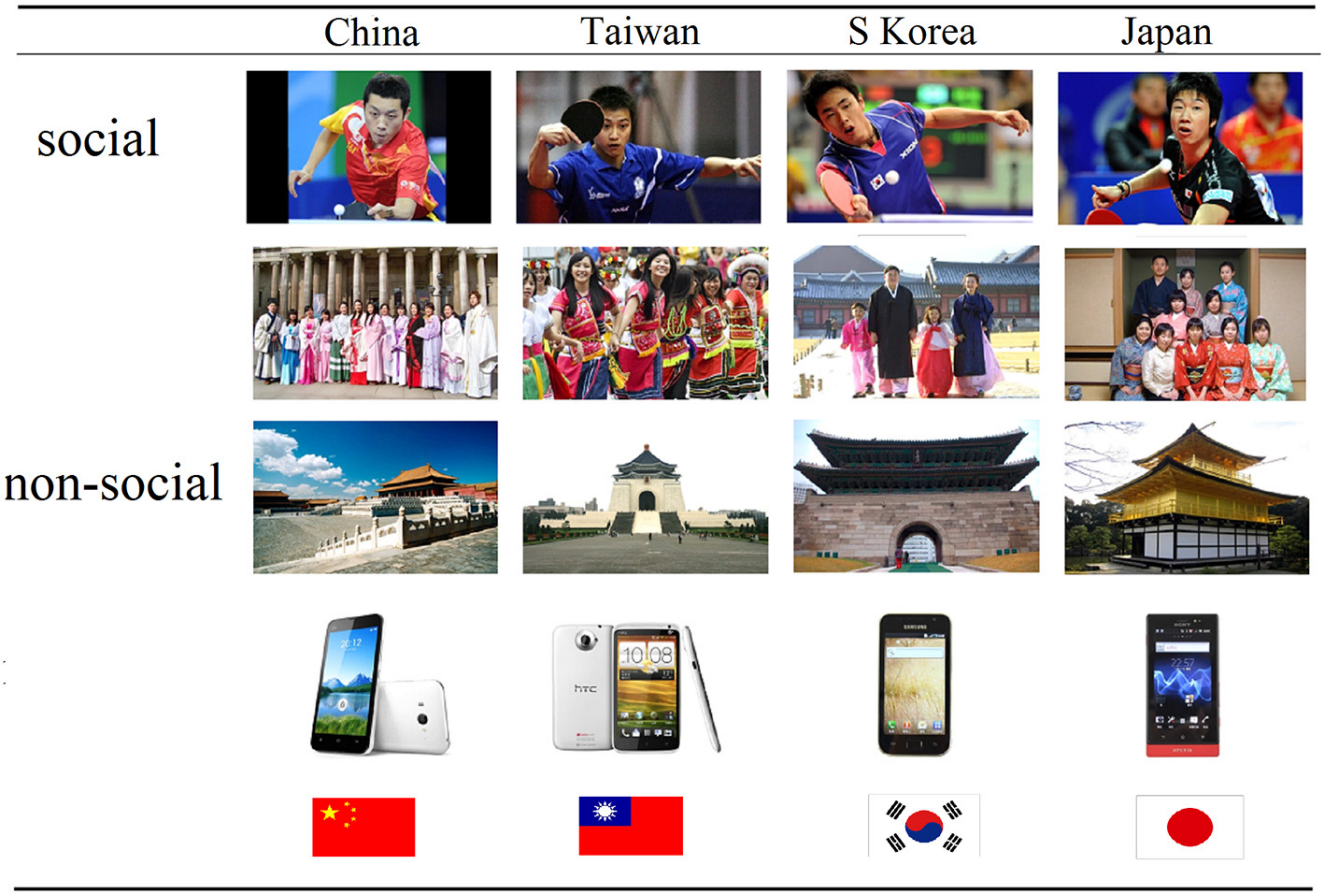
**Examples of stimuli: social and non-social Chinese, Taiwanese, Japanese, and South Korean stimulus pictures used in the study**. The bottom row of pictures (mobile phones) also shows the size of the country flag which appeared under each of the stimuli presented at aid identification of Nationality.

The stimulus pictures used in the study were all taken from the internet and both social and non-social item categories used were deliberately chosen as being likely to evoke feelings of National pride. Social category stimulus pictures either depicted individuals, groups or names (*n* = 48) whereas non-social category ones depicted iconic symbols or consumer products/companies from four different countries (China, Taiwan, Japan, and South Korea) (*n* = 48). The 96 pictures were displayed in a random order for 3 s each and with a further 3 s for each rating question to be answered using a displayed 9 point scale. There was a 1 s period where a white fixation cross was displayed on a black background (jittered 0.5–1.5 s) before each stimulus picture. Stimulus presentations were made using E-prime software. For the social and non-social stimuli 12 different pictures from 5 different categories (Political leader; sports men and women; school children; people wearing typical national dress in different contexts and popular given names were used (Social—1: National leader, 2: Olympic gold-medal champion—male, 3: Olympic gold-medal champion—female, 4: National table-tennis player—male, 5: National table-tennis player—female, 6: National male football team, 7: school children in uniform, 8: Man and woman in traditional wedding dress; 9: Two martial arts players fighting—male, 10: Men and women in traditional opera dress, 11: Men and women in traditional dress; 12: Popular given names. Non-social—1: National flag, 2: National Flag-map, 3: Picture of capital city, 4: Ancient iconic building, 5: Modern iconic building, 6: Iconic monument, 7: Bank note (100RMB and equivalent value for other countries), 8: Traditional food, 9: Famous supermarket brand; 10: Mid-range value car with manufacturer logo, 11: Economy car with manufacturer logo; 12: Similar value touch-screen mobile telephones showing manufacturer name). To aid subjects in identifying the country represented in the pictures all of them had a small national flag inserted underneath the picture displayed. In all cases it was first confirmed that subjects used were highly familiar with the flags of the 4 countries. Examples of stimulus pictures are shown in Figure 1.

We first used an independent cohort of 20 subjects (*n* = 10 female) to rate Chinese social and non-social pictures on a scale of 1–9 for (1) likeability and also for the questions: (2) How much does this picture make you feel that China is better than other countries and (3) How much does this picture make you proud to be Chinese? Results showed that all the Chinese social and the majority of non-social (the two unsurprising exceptions were money and food) picture categories chosen evoked equivalent high scores on all three questions [mean ± s.e.m. rating scores—Q1: Social 6.95 ± 0.29, Non-social 7.31 ± 0.27, *t*_(22)_ = 0.95, *p* = 0.36; Q2: Social 6.75 ± 0.26, Non-social 6.80 ± 0.24, *t*_(22)_ = 0.13, *p* = 0.90; Q3: Social 6.99 ± 0.31, Non-social 6.96 ± 0.22, *t*_(22)_ = 0.057, *p* = 0.96] showing that the Chinese social and non-social stimulus sets did not differ significantly in their likeability or their ability to generate feelings of National pride. Furthermore, with the exception of two non-social pictures (money and food) there was a high positive correlation between likeability ratings and ratings given by individual subjects on both questions about feelings of evoked national pride for both the social (overall mean correlation of 0.77 ± 0.03 for Q2 and 0.68 ± 0.05 for Q3) and non-social pictures (0.69 ± 0.04 for Q2 and 0.65 ± 0.05 for Q3). We therefore decided for the main experiment to ask subjects to provide only likeability ratings to avoid deliberately priming them to consider their feelings of National pride. For the first part of the main experiment (test 1) subjects were seated in front of a computer screen and asked to rate the social and non-social pictures from the four countries on a scale of 1–9 for likeability (1 = dislike very much, 5 = neutral and 9 = like very much) as well as for arousal (1 = not aroused at all and 9 = very aroused) and familiarity (1 = completely unfamiliar and 9 = very familiar). Subjects were shown and required to rate the 96 different pictures presented in a random order and told that their scores would be kept anonymous.

For the second part of the experiment (test 2) subjects were asked to return 7 days later in order to take part in a completely different experiment. However, instead when they returned they were first re-administered the PANAS questionnaire (to ensure that they had not experienced a significant mood change during the week between the tests) and then unexpectedly asked to perform the original task again, although with stimuli being presented in a different random order. All original 51 subjects completed this second test and no additional OXT or PLC treatment was given. No studies have reported significant time of day effects for intranasal OXT treatment in humans and so we did not restrict tests to a specific time of day. In both experiments subjects were tested between 09.00 and 20.00 (mainly 14.00–16.00) and there were no overall differences between the treatment groups, or between tests 1 and 2 within groups (*p* > 0.1 in all cases). On average differences in test times for individual subjects in test 1 and test 2 were <2 h.

## Statistics

Likeability, arousal and familiarity ratings made by subjects were averaged separately across the 12 social and 12 non-social stimulus pictures for each of the four different countries. In all cases mixed factor repeated measure ANOVA tests were performed using SPSS 17.0 (SPSS Inc., Chicago, IL, USA) with significant interaction effects being explored using Simple Effect Tests and corrected for multiple comparisons using Bonferoni. In the main analysis treatment and sex were considered as between-subject factors and country, social vs. non-social and test 1 vs. test 2 as within-subject factors. Where secondary ANOVA analyses were carried out separately for the social and non-social stimuli and including stimulus sub-categories as a within-subject factor this was corrected for by reducing the accepted significance level for main effects and interactions to *p* < 0.025. No correction was applied to *p*-values for additional ANOVAs investigating potential treatment effects on stage of menstrual cycle, arousal or familiarity since these were considered as controls and not predicted to reveal significant treatment effects.

## Results

Table 1 shows that there were no significant differences between the OXT and PLC groups in age or for personality, empathy, anxiety, or depression scores. There were also no significant differences between scores on the PANAS administered prior to each of the two tests.

**Table 1:** **Ages and questionnaire scores for study subjects (mean ± s.e.m.)**.

**Measurements**	**Placebo**	**Oxytocin**	***t*-value**	***p*-value**	**CI^a^**
Age (years)	22.0 ± 0.3	22.3 ± 0.3	−0.86	0.395	−1.3 to 0.5
**NEO-FIVE FACTOR INVENTORY (NEO-FFI)**					
Neuroticism	34.4 ± 2.1	33.0 ± 1.4	0.56	0.577	−3.6 to 6.3
Extraversion	39.4 ± 1.3	40.5 ± 1.3	−0.62	0.541	−4.7 to 2.5
Openness to experience	39.5 ± 1.1	40.0 ± 1.2	−0.28	0.781	−3.8 to 2.9
Agreeableness	40.3 ± 1.1	41.7 ± 0.8	−1.05	0.301	−3.9 to 1.2
Conscientiousness	40.9 ± 1.1	42.7 ± 1.1	−1.15	0.256	−5.0 to 1.4
Self-Esteem Scale (SES)	33.0 ± 0.9	33.6 ± 0.7	−0.61	0.548	−2.9 to 1.6
Empathy Quotient (EQ)	36.4 ± 1.4	37.0 ± 1.5	−0.32	0.752	−4.8 to 3.5
Beck Depression Inventory (BDI-II)	8.3 ± 1.5	9.5 ± 1.2	−0.64	0.524	−5.1 to 2.6
State-Trait Anxiety Inventory (STAI)—State	42.0 ± 2.0	37.6 ± 1.7	1.69	0.097	−0.8 to 9.7
State-Trait Anxiety Inventory (STAI)—Trait	43.8 ± 2.0	40.1 ± 1.8	1.40	0.167	−1.6 to 9.1
**POSITIVE AND NEGATIVE AFFECTIVE SCALE (PANAS)—POSITIVE**					
Test 1	27.9 ± 1.5	29.3 ± 1.0	−0.81	0.424	−5.1 to 2.2
Test 2	27.1 ± 1.5	26.5 ± 2.0	0.21	0.831	−4.5 to 5.5
**POSITIVE AND NEGATIVE AFFECTIVE SCALE (PANAS)—NEGATIVE**					
Test 1	19.0 ± 1.3	17.8 ± 1.1	0.76	0.453	−2.1 to 4.6
Test 2	16.9 ± 1.1	14.9 ± 1.6	1.04	0.307	−1.9 to 5.9

a *95% Confidence interval*.

**Figure 2:**
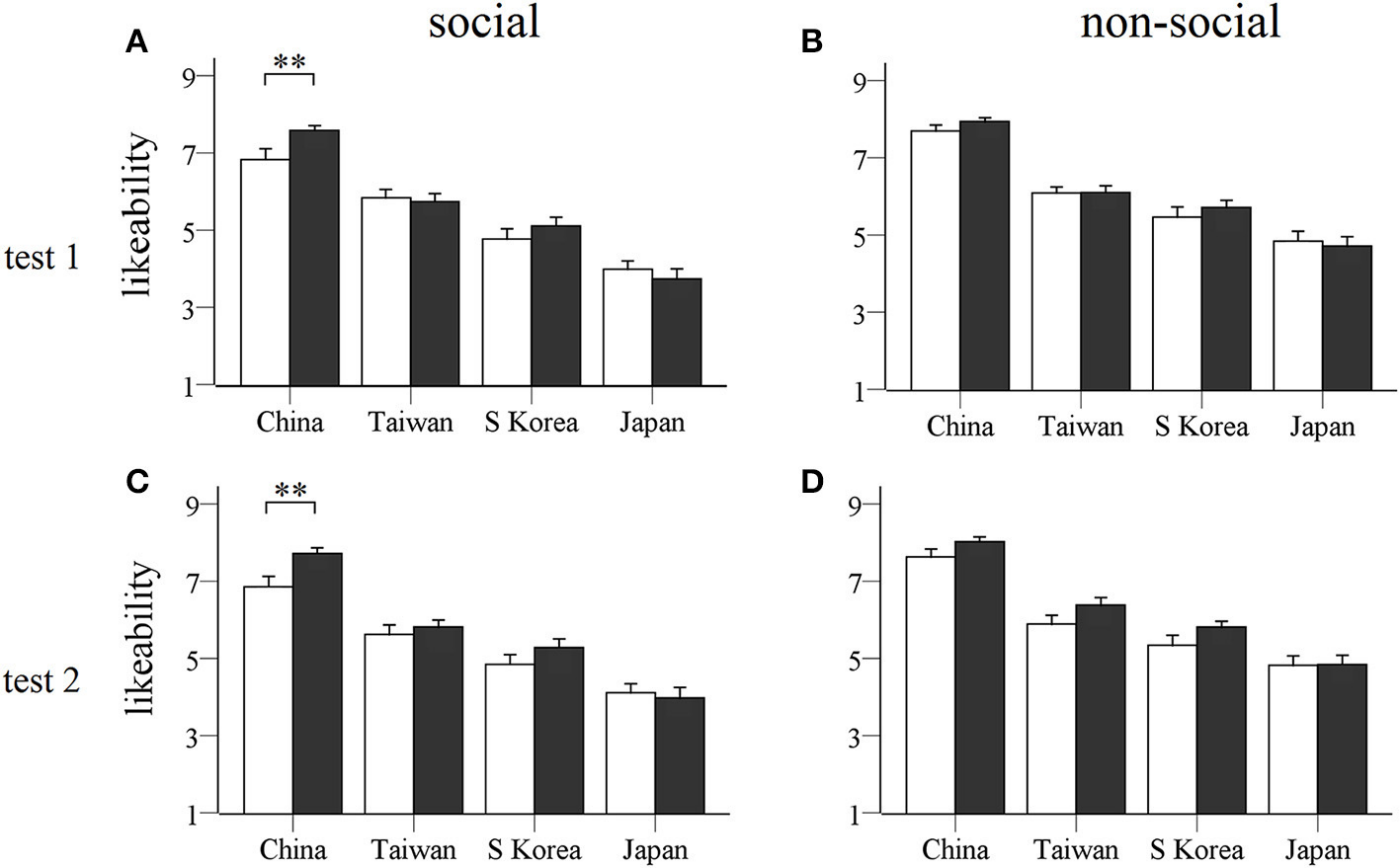
**Likeability ratings for each country**. Histograms show mean ± s.e.m. likeability ratings of social **(A,C)** and non-social **(B,D)** stimulus pictures for four different countries given by OXT (filled bars) and PLC (unfilled bars) treated subjects in the two tests (**A,B**: test 1, 45 min after OXT/PLC treatment; **C,D**: test 2, 7 days after treatment). ^**^p < 0.01 for OXT vs. PLC.

For likeability scores an ANOVA revealed main effects of social vs. non-social stimuli [*F*_(1, 47)_ = 61.60, *p* < 0.0001, η^2^ = 0.576] and country [*F*_(3, 141)_ = 138.53, *p* < 0.0001, η^2^ = 0.747] and a significant interaction between social vs. non-social stimuli and country [*F*_(3, 141)_ = 6.18, *p* = 0.002, η^2^ = 0.116]. Figure 2 shows this was due to the non-social stimuli being scored significantly higher than social ones for all four countries and the expected ethnocentric effect with overall score for both social and non-social stimuli being highest for China followed by Taiwan and then South Korea with Japan the lowest. *Post-hoc* comparison tests revealed likeability scores for Chinese picture stimuli were significantly higher than all three other nations during both tests (*p* < 0.0001 for both social and non-social); scores for Taiwanese pictures were significantly higher than for South Korean and Japanese ones (*p* < 0.01) and scores for South Korean pictures were significantly higher than for Japanese ones (*p* < 0.0001). There was a significant interaction between social vs. non-social × country × sex [*F*_(3, 141)_ = 2.90, *p* = 0.037, η^2^ = 0.058] which was contributed to by a slightly higher likeability ratings for Chinese and Taiwanese non-social stimuli in male subjects and lower ratings for Japanese social stimuli in female subjects, although none of these differences achieved significance (*p* > 0.176 in all cases). Most importantly in the context of the current study there was also a significant interaction between treatment × social vs. non-social × country [*F*_(3, 141)_ = 4.26, *p* = 0.007, η^2^ = 0.083] which Figure 2 shows is due to OXT increasing likeability scores for Chinese social, but not non-social stimuli in both tests (OXT vs. PLC for social stimuli from China, test 1: *p* = 0.0096; test 2: *p* = 0.0065). There was no overall significant difference between likeability scores given by subjects in test 1 vs. test 2 (China social: PLC – *p* = 0.76, OXT – *p* = 0.50; China non-social: PLC – *p*= 0.33, OXT – *p*= 0.77). OXT had no significant effect on social stimuli from other countries (test 1: Taiwanese, *p* = 0.745; Japanese, *p* = 0.455; South Korean, *p* = 0.292; test 2: Taiwanese, *p* = 0.510; Japanese, *p* = 0.708; South Korean, *p* = 0.177). There were no significant differences between likeability scores for the social pictures from these 3 countries in test 1 vs. test 2 (*p* >0.1 in all cases). There was however a test × treatment × sex interaction [*F*_(3, 141)_ = 5.80, *p* = 0.020, η^2^ = 0.110]. This was due to a slight but consistent reduction in overall likeability ratings given by female subjects to both social and non-social stimuli across all four countries in the PLC group in test 2 compared with test 1 (*p* = 0.015). On the other hand likeability ratings in OXT-treated subjects were very similar in tests 1 and 2. In males ratings in both PLC and OXT treated individuals were similar in tests 1 and 2.

To control for the possibility of a potential interaction between stage of menstrual cycle and OXT effects on likeability ratings we performed a separate ANOVA on test 1 scores in female subjects in the OXT and PLC groups and including stage of cycle as a factor (Follicular vs. Luteal – OXT group *n* = 4 follicular and *n* = 9 luteal; PLC group *n* = 3 follicular and *n* = 10 luteal). Results showed there was neither a main effect of cycle phase [*F*_(1, 22)_ = 0.534, *p* = 0.473, η^2^ = 0.024] nor an interaction between treatment × cycle phase [*F*_(1, 22)_ = 0.251, *p* = 0.621, η^2^ = 0.011].

**Figure 3:**
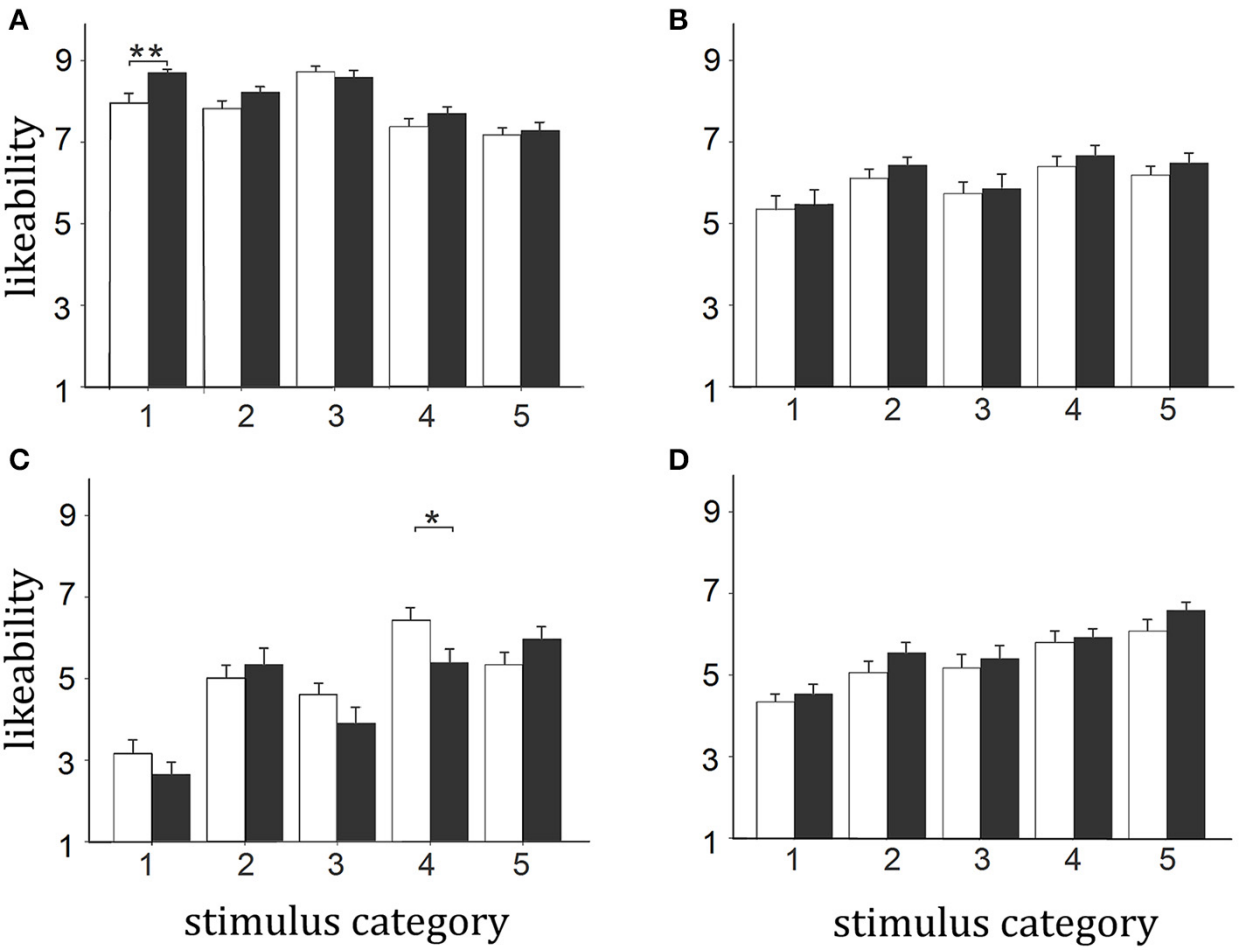
**Likeability ratings of different non-social stimulus categories for: (A) Chinese, (B) Taiwanese, (C) Japanese, and (D) South Korean**. Histograms show overall mean ± s.e.m. combined likeability ratings for the different stimulus picture categories given by OXT and PLC treated subjects in the tests given 45 min (test 1) and 7 days (test 2) after treatment. Non-social categories—1. Chinese flag – Flag and Flag-map; 2. Ancient and modern buildings and monuments and Capital cities—capital city, ancient iconic building, modern iconic building and monument; 3. Money—100RMB or equivalent Bank note; 4. Traditional national food and food supermarket; 5. Consumer products and their manufacturers—mid-range value car with manufacturer logo, economy car with manufacturer logo, touch-screen mobile telephone showing manufacturer name. ^**^p < 0.01 and ^*^p < 0.05 for OXT vs. PLC.

In view of the fact that both social and non-social stimulus pictures contained a number (*n* = 5 for both social and non-social) of distinct sub-categories, and we hypothesized OXT might have had differential effects on these due to contextual experience, we performed a second analysis on tests 1 and 2 including these sub-categories as a within-subject factor. For social stimuli the sub-categories used were: (1) Party Leader picture 1, (2) Famous sports stars – pictures 2–6, (3) School children in typical uniform – picture 7, (4) Adults taking part in events in traditional dress – pictures 8–11, (5) Popular given names – picture 12; for non-social stimuli there were: (1) The National flag – pictures 1,2, (2) Ancient and Modern National monuments and buildings – pictures 3–6, (3) Money – picture 7, (4) Typical national food and food supermarket – pictures 8,9 and (5) Consumer products from National companies – pictures 10–12. An initial analysis revealed no significant differences between scores for the different categories in test 1 vs. test 2 and so scores from the 2 tests were combined. Separate ANOVAs were therefore performed for social and non-social category sub-groups with categories (*n* = 5) and country as within group factors and treatment and sex as between subject factors. For the social stimuli there was a main effect of category [*F*_(4, 47)_ = 22.67, *p* < 0.0001, η^2^ = 0.325] and country [*F*_(3, 47)_ = 111.79, *p* < 0.0001, η^2^ = 0.704] and a category × country interaction [*F*_(12, 47)_ = 13.92, *p* < 0.0001, η^2^ = 0.229]. This showed that there were differences between the likeability ratings of the 5 different stimulus categories both within and across countries. While there was no treatment × category interaction [*F*_(4, 47)_ = 0.169, *p* = 0.954, η^2^ = 0.004], indicating that OXT effects were similar across the five categories, there was a marginally significant treatment × country interaction [*F*_(3, 47)_ = 2.89, *p* = 0.038, η^2^ = 0.058 – *p* < 0.025 considered significant following correction for multiple ANOVAs] due to OXT effects occurring mainly for Chinese stimuli (*p* = 0.014). There were no other significant main effects or interactions. For the non-social stimulus categories there was a similar pattern with main effects of category [*F*_(4, 47)_ = 25.05, *p* < 0.0001, η^2^ = 0.348] and country [*F*_(3, 47)_ = 151.52, *p* < 0.0001, η^2^ = 0.763] and a category × country interaction [*F*_(12, 47)_ = 34.02, *p* < 0.0001, η^2^ = 0.420]. While there was no treatment × category interaction [*F*_(4, 47)_ = 1.528, *p* = 0.196, η^2^ = 0.031], there was a significant treatment × country × category one [*F*_(12, 47)_ = 2.13, *p* = 0.014, η^2^ = 0.043]. *Post-hoc* tests corrected for multiple comparisons showed that this was due to OXT significantly increasing likeability ratings for the Chinese flag stimulus category (*p* = 0.005) and decreasing them for the Japanese food/food supermarket category (*p* = 0.029) (see Figure 3). No other significant differences were found across the stimulus categories from the different nations (*p* > 0.1 in all cases). There were no other significant main effects or interactions.

**Figure 4:**
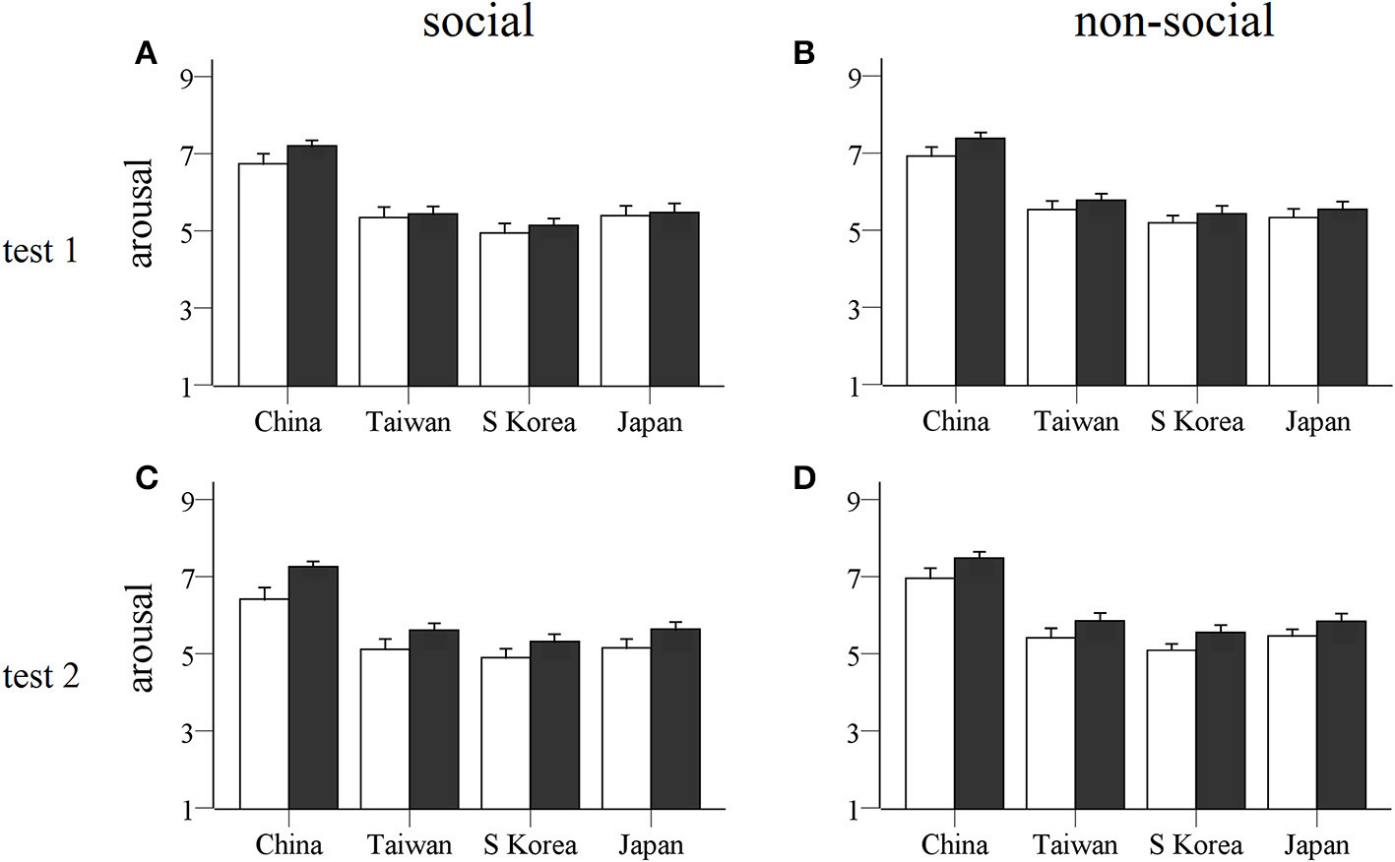
**Arousal ratings for stimulus pictures from each country**. Histograms show mean ± s.e.m. arousal ratings of social **(A,C)** and non-social **(B,D)** stimulus pictures for four different countries given by OXT (filled bars) and PLC (unfilled bars) treated subjects in the two tests (**A,B**: test 1, 45 min after OXT/PLC treatment; **C,D**: test 2, 7 days after treatment). No significant treatment effects were found.

For arousal scores (see Figure 4) there were also significant main effects of social vs. non-social stimuli [*F*_(1, 47)_ = 13.82, *p* = 0.001, η^2^ = 0.227] and country [*F*_(3, 141)_ = 86.15, *p* < 0.0001, η^2^ = 0.647]. This suggests that the non-social picture stimuli were significantly more arousing than the social ones, with highest arousal ratings for Chinese followed by Japanese and Taiwanese, with South Korean ones evoking the lowest arousal. There were no significant treatment interaction effects and so, as predicted, there was no evidence for an overall effect of OXT on arousal ratings. Similarly, in the secondary ANOVA analysis including the five different categories of social and non-social stimuli there were no significant treatment × category, treatment × country or treatment × country × category interactions (*p* > 0.66 in all cases) confirming that OXT did not influence arousal responses for any specific stimulus category.

**Figure 5:**
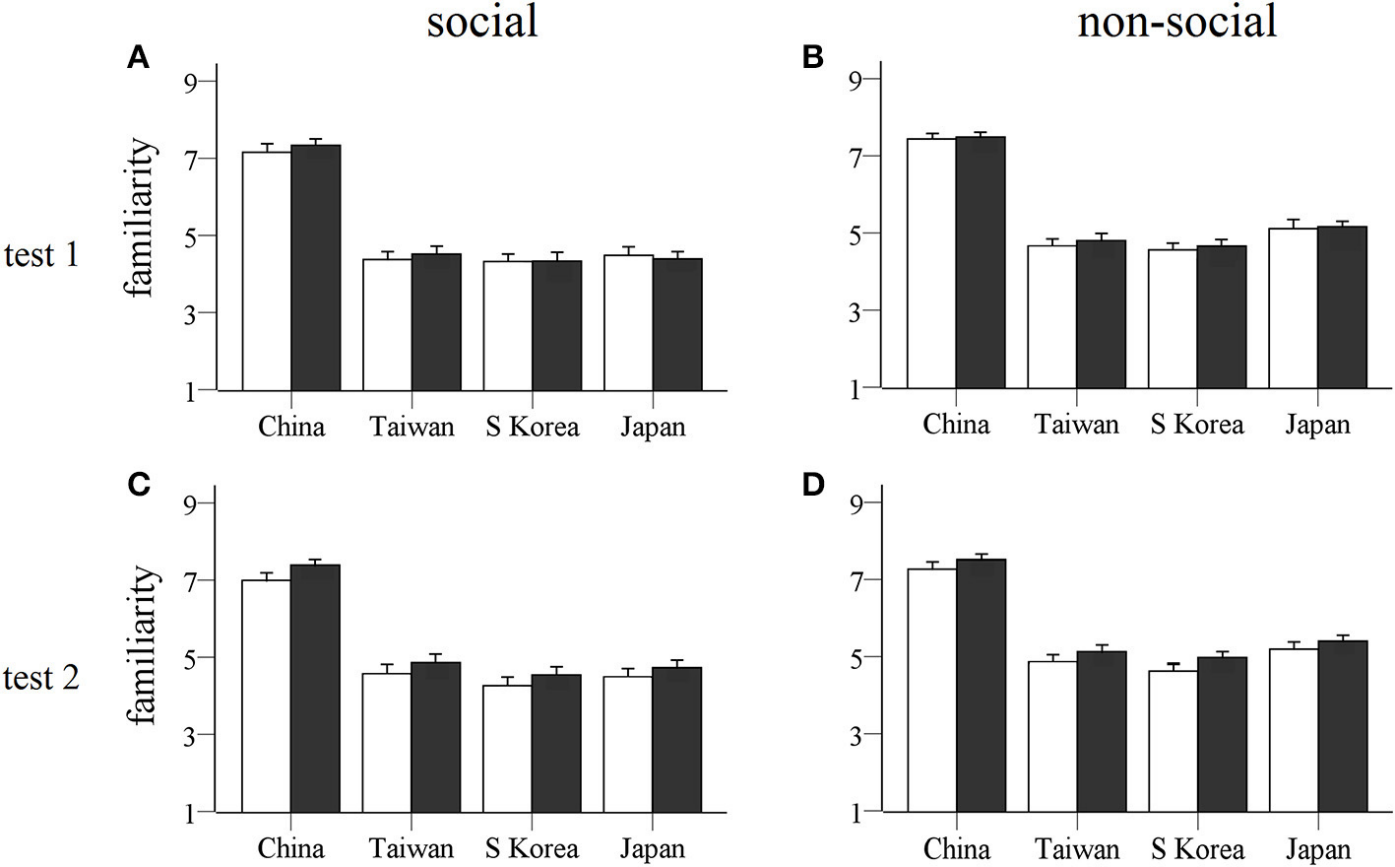
**Familiarity ratings for stimulus pictures from each country**. Histograms show mean ± s.e.m. familiarity ratings of social **(A,C)** and non-social **(B,D)** stimulus pictures for four different countries given by OXT (filled bars) and PLC (unfilled bars) treated subjects in the two tests (**A,B**: test 1, 45 min after OXT/PLC treatment; **C,D**: test 2, 7 days after treatment). No significant treatment effects were found.

Analysis of familiarity scores (see Figure 5) also revealed a significant main effect of social vs. non-social stimuli [*F*_(1, 47)_ = 33.84, *p* < 0.0001, η^2^ = 0.419] and country [*F*_(3, 141)_ = 318.82, *p* < 0.0001, η^2^ = 0.872] and a significant interaction between social vs. non-social stimuli and country [*F*_(3, 141)_ = 8.25, *p* < 0.0001, η^2^ = 0.149]. This showed that the non-social stimuli were scored significantly higher than social ones for picture stimuli from all four countries (*p* < 0.01 in all cases) and that, as expected, subjects were most familiar with Chinese social and non-social stimuli (*p* < 0.0001), followed by Taiwanese, Japanese, and South Korean for social stimuli, or Japanese, Taiwanese, and South Korean for non-social stimuli. There was a significant test × country interaction [*F*_(3, 141)_ = 3.81, *p* = 0.012, η^2^ = 0.075]. This was mainly contributed to by subjects rating Taiwanese picture items more familiar in test 2 compared to test 1 (*p* = 0.010). There was also a trend for a test × treatment × sex interaction [*F*_(3, 141)_ = 3.76, *p* = 0.059, η^2^ = 0.074] with the *post-hoc* analysis showing that males in the OXT group generally rated pictures as significantly more familiar in test 2 compared with test 1 (*p* = 0.02). Similarly in the secondary ANOVA analysis including the 5 different categories of social and non-social stimuli there were no significant treatment × category, treatment × country or treatment × category × country interactions (*p* > 0.224 in all cases) showing that, as expected, OXT did not influence familiarity responses for any specific stimulus category.

Although, as predicted, we found no significant OXT effects on arousal and familiarity ratings we nevertheless also explored their potential contributions to main finding of an OXT effect on likeability scores in the two different tests. We therefore also performed separate ANOVAs on test 1 and test 2 including arousal and familiarity scores as nuisance covariates. This analysis confirmed that there was still a significant, albeit reduced, treatment × social vs. non-social × country interaction in both tests [test 1: *F*_(3, 141)_ = 2.80, *p* = 0.043, η^2^ = 0.058; test 2: *F*_(3, 141)_= 2.66, *p* = 0.051, η^2^ = 0.056]. *Post-hoc* comparison tests also confirmed a significant increase in likeability scores for China social stimuli in the OXT group in test 1 (*p* = 0.02) and a trend toward significance in test 2 (*p* = 0.077). Thus, while an OXT effect on arousal and familiarity may have contributed to some extent to increased likeability scores for the Chinese social stimuli in both tests, it also appears to have significant effects independent of them.

## Discussion

Overall our results have shown that intranasal OXT facilitates likeability ratings in both male and female Chinese subjects in terms of pictures depicting Chinese politicians, sports men and women, men and women in traditional dress and school children compared with those associated with other Asian countries (Taiwan, South Korea, and Japan). On the other hand OXT treatment had no overall effect on similar liking for national and cultural symbols (such as food, money, cities, buildings, and monuments) and consumer outlets/products (supermarkets, cars, and mobile phones). A further analysis which divided stimulus pictures into different sub-categories revealed that an exception to this was the two pictures (flag and flag-map) showing the Chinese national flag where there was also a clear facilitatory effect of OXT on likeability ratings. Despite clear evidence for derogation (in terms of lower likeability ratings) of both social and non-social pictures depicting out-group cultures (notably Japanese where average scores were mainly in the “dislike” rather than “like” part of the scale) there was no overall evidence that this was influenced by OXT, other than a significant reduction in combined ratings for a typical Japanese food dish (sushi) and food supermarket (Ito Yokado). There were no significant effects of OXT on arousal or familiarity ratings on either the social or non-social picture sets from the four different countries. Importantly, we have also shown that these behavioral effects of OXT were maintained even a week after treatment. Thus, our results have also demonstrated for the first time that behavioral effects of OXT treatment can be maintained for some time following treatment and are not restricted to the immediate period of elevated concentrations after intranasal application.

While overall increases in likeability scores for Chinese social stimuli following OXT treatment were relatively small they were highly consistent across the different sub-categories of stimuli. The strongest effects were seen for pictures of men and women in various forms of traditional dress and for sports stars, followed by the current party Chairman (Chairman Xi), with only popular names failing to show a significant effect. Considering the Chinese social stimuli were given high average likeability ratings in the PLC treated group (mean = 6.88/9), it is interesting that OXT treatment was still able to increase them further (mean = 7.69/9). The majority of previous studies reporting similar magnitude OXT effects on aspects of social attraction have tended to use stimuli with relatively neutral initial valence (Theodoridou et al., 2009; Striepens et al., 2014).

There was clearly no overall effect of OXT on ethnocentric attitudes underlying the “country of origin” effect for commercial products and their manufacturers, and also no effect in relation to ancient/modern buildings/monuments or money. There was a slightly greater liking for non-social rather than social pictures and so the absence of an effect of OXT on them is unlikely to have been because they were less interesting. Since likeability ratings were very high for most of the non-social stimuli it is possible OXT effects might have been obscured by a potential ceiling effect, although there was only a small difference in ratings compared with social stimuli where robust effects did occur. Also, there was a strong significant facilitatory action of OXT for increasing likeability of pictures of the National flag (the flag alone and a flag-map) despite the high ratings observed in the PLC group. Previous studies have shown that National flags are a potent and influential stimulus where even subliminal exposure can influence political thought and behavior (Hassin et al., 2007) and in-group normative values (Sibley et al., 2011). Since National flags are often carried or worn by people this may represent a contextual situation where a non-social stimulus can effectively become equivalent to a social one as a result of repeated social associations. While it is also possible that OXT is not simply acting on social stimuli *per se* in this context but is instead increasing an individual's sense of National affiliation and pride, the social and non-social picture sets used in our study received equivalent ratings for evoking feelings of National pride by a group of independent raters. Although the two flag pictures used did elicit high National pride ratings other national iconic symbols such as Beijing and the Forbidden City were rated even higher. Thus, OXT effects are unlikely to be specifically associated with levels of National pride evoked by social or non-social picture stimuli.

All the picture stimuli used in the current study included a small national flag underneath them in order to guarantee that subjects correctly identified the country of origin and it is possible that this may have contributed to the observed ethnocentric effects for both social and non-social items. However, while OXT facilitated likeability ratings for the main picture stimuli depicting the Chinese National flag it is clear that this effect did not extend to the smaller flag icon positioned underneath the target pictures. If this were the case then it would be expected that all social and non-social picture items would have shown a facilitatory OXT effect, which clearly they did not. Thus, it seems likely that OXT effects were restricted primarily to the target stimulus images which would have been the main focus of attention.

In line with the majority of studies which have reported OXT effects on behavior toward “in-group” or “out-group” members (see recent meta-analysis by Van IJzendoorn and Bakermans-Kranenburg, 2012), we mainly found evidence for increased “in-group” favoritism and not additionally for increased “out-group” derogation. Indeed, the only somewhat indirect evidence for the latter was in terms of significantly reduced liking for food-related stimuli (Japanese sushi and the food supermarket Ito Yokado). However, while some consider ethnocentrism to involve both in-group bias and favoritism as well as out-group derogation, recent research has suggested that these two components of ethnocentrism are separable constructs (Bizumic and Duckitt, 2012) and therefore OXT may specifically be acting on the in-group favoritism factor rather than out-group derogation.

To the best of our knowledge this is the first study to investigate potential gender differences in the context of OXT effects on “in group” favoritism/ethnocentrism. Despite growing evidence for gender differences for OXT actions on physiological and emotional responses (Kubzansky et al., 2012; Ditzen et al., 2013; Fischer-Shofty et al., 2013; Rilling et al., 2014; Hoge et al., 2014; Yao et al., 2014) we found no clear evidence for this in the context of the current study. A recent study reporting OXT-induced increases in empathy for pain in out-group individuals also found no gender difference in responses to OXT (Shamay-Tsoory et al., 2013). While we did not control specifically for possible menstrual cycle effects we did not find any main effects of the follicular vs. luteal phase or any interaction between treatment and cycle phase. Several other studies have also failed to find an influence of the menstrual cycle on functional effects of OXT treatment (Theodoridou et al., 2009; Domes et al., 2010). Nevertheless, it is possible some subtle differences would have been found if we had used a larger subject group or a more extensive range of stimulus pictures, although the current study design was deliberately constructed to include pictures that had an overall similar attraction for both males and females.

Our finding that likeability for Chinese social and flag pictures was maintained for 1 week after intranasal OXT treatment suggests that behavioral effects induced during experience of increased concentrations can be maintained even after they have declined. It is possible that OXT may have either facilitated learning of the increased likeability ratings given to specific stimuli during test 1 which resulted in the same higher ratings being given in test 2. Alternatively, or additionally, OXT may have produced a more general increase in likeability of Chinese social stimuli and flags. While our study design cannot distinguish between these two possibilities it is interesting that female subjects in the PLC group showed a significant decline in likeability ratings in test 2 compared to test 1 across all the different nations while this was not the case in the OXT group. Thus, for female subjects at least ratings produced in test 1 were not simply learned and reproduced in test 2 and that instead a revised likeability judgment occurred. In the female OXT group on the other hand maintenance of similar likeability ratings may either have been the result of stronger learning of the initial rating given, or because subjects did not want to revise their judgment. Animal studies for example have demonstrated that under conditions where OXT concentrations are elevated both neural plasticity and learning effects (Kendrick, 2000; Ferri and Flanagan-Cato, 2012) can occur, particularly in the context of social recognition. In humans intranasal OXT also facilitates learning with social feedback (Hurlemann et al., 2010). Nevertheless, although we cannot be sure of the precise mechanism whereby OXT-induced changes in likeability ratings were maintained for a week post-treatment in the current experiment it is clear that in certain contexts behavioral preferences can remain altered well-beyond the immediate period of elevated concentrations following intranasal treatment. This may have important implications for potential therapeutic-treatment protocols using intranasal OXT treatment where daily dosing regimens are typically been used. In some instances less frequent treatments when combined with behavioral training might also be effective in influencing long-term behavioral changes and reduce the risk of possible receptor desensitization problems. There is evidence for increased CSF concentrations of OXT following intranasal application in humans (Striepens et al., 2013) and monkeys (Chang et al., 2012), and other similar peptides in humans (Born et al., 2002), and it is generally considered that OXT given intranasally crosses the blood-brain barrier to penetrate into the brain cerebrospinal system (see Striepens et al., 2011, 2013; Neumann et al., 2013). The duration of elevated OXT concentrations in CSF following intranasal treatment has not been clearly established, although findings reported in recent *in vivo* microdialysis study in rats (Neumann et al., 2013) and on endogenous release profiles in sheep following birth (Kendrick et al., 1991) suggest that they are unlikely to persist for more than a matter of a few hours.

Oxytocin had no significant effect on arousal and familiarity ratings for the picture stimuli used in the two tests, and OXT effects on likeability ratings were still present even when they were included as nuisance covariates, although significance levels were reduced. It is therefore unlikely that increased liking for Chinese social and flag pictures in either of the tests was contributed to primarily by enhanced arousal responses or making items appear more familiar. This is in agreement with a number of other studies which have also reported no effect of OXT on arousal (Scheele et al., 2012; Striepens et al., 2012, 2014). However, we cannot entirely rule out the possibility that some contribution of arousal and familiarity to increased likeability following OXT treatment might have occurred.

Studies showing OXT enhancement of attractiveness ratings of faces have found associated increases in activity in striatal reward regions (Scheele et al., 2013; Striepens et al., 2014) and so it is possible that in the present study increased liking for Chinese picture stimuli was also associated with OXT promoting their rewarding effect. This possibly requires experimental confirmation however.

A limitation of the current study was that relatively few picture stimuli were used for each individual country and possibly a larger number and wider range of stimuli might have revealed additional effects. We deliberately included a number of different categories of both social and non-social and our analysis did reveal some evidence for differential effects of OXT. Another potential limitation was that experiments were conducted across a wide time range, in terms of time of day, and this may have had some influence on results. However, although there may be circadian variations in endogenous CSF OXT concentrations no previous studies have reported evidence for time of day effects on behavioral effects of intranasal OXT treatment. Importantly there were no significant differences between the treatment groups and on average re-tests after 1 week were conducted within 2 h of the time of the original one. A final limitation, as discussed above, was that our paradigm did not allow us determine whether maintenance of altered likeability ratings 1 week after OXT treatment reflects a generalized revised liking for Chinese stimuli or a facilitation of learning of the original ratings given for the specific stimuli.

In summary, the current study has shown a facilitatory effect of OXT on ethnocentrism in both men and women in terms of increased liking for pictures depicting both social and flag stimuli from the subjects' native country, China. There was no strong evidence for an influence of OXT on likeability of either social or non-social stimuli from other Asian countries despite clear general evidence for dislike of some out-group stimuli. The behavioral effects of a single intranasal dose of OXT were maintained for at least a week.

### Conflict of interest statement

The authors declare that the research was conducted in the absence of any commercial or financial relationships that could be construed as a potential conflict of interest.
